# miR-452-3p inhibited osteoblast differentiation by targeting Smad4

**DOI:** 10.7717/peerj.12228

**Published:** 2021-09-28

**Authors:** Ming Wu, Hongyan Wang, Dece Kong, Jin Shao, Chao Song, Tieyi Yang, Yan Zhang

**Affiliations:** 1 Postgraduate Training Base in Shanghai Gongli Hospital, Ningxia Medical University, Shanghai, China; 2 Department of Orthopaedics, Gongli Hospital of Pudong New Area, Shanghai, China

**Keywords:** MC3T3-E1, miR-452-3p, Smad4, Osteoporosis, Osteoblast differentiation

## Abstract

Osteoblast differentiation is a complex process that is essential for normal bone formation. A growing number of studies have shown that microRNAs (miRNAs) are key regulators in a variety of physiological and pathological processes, including osteogenesis. In this study, BMP2 was used to induce MC3T3-E1 cells to construct osteoblast differentiation cell model. Then, we investigated the effect of miR-452-3p on osteoblast differentiation and the related molecular mechanism by RT-PCR analysis, Western blot analysis, ALP activity, and Alizarin Red Staining. We found that miR-452-3p was significantly downregulated in osteoblast differentiation. Overexpression miR-452-3p (miR-452-3p mimic) significantly inhibited the expression of osteoblast marker genes RUNX2, osteopontin (OPN), and collagen type 1 a1 chain (Col1A1), and decreased the number of calcium nodules and ALP activity. In contrast, knockdown miR-452-3p (miR-452-3p inhibitor) produced the opposite effect. In terms of mechanism, we found that Smad4 may be the target of miR-452-3p, and knockdown Smad4 (si-Smad4) partially inhibited the osteoblast differentiation enhanced by miR-452-3p. Our results suggested that miR-452-3p plays an important role in osteoblast differentiation by targeting Smad4. Therefore, miR-452-3p is expected to be used in the treatment of bone formation and regeneration.

## Introduction

Osteoporosis is a globally prevalent bone condition connected with bone resorption and loss of the bone microstructure, which can lead to bone fragility and increased bone fracture (Learmonth, Young & Rorabeck, 2007). Therefore, one of the key methods to manage the imbalance in bone mass is to stimulate osteoblast formation (Oh et al., 2019). Osteoblasts serve critical functions in both early bone formation and subsequent bone remodelling processes (Ruan et al., 2017). Bone formation involves the differentiation of progenitor cells into osteoblasts (Raic et al., 2019); Inhibiting this process may have pathological consequences (Liu et al., 2020b). Osteoblasts are important for bone production, and some protein markers, such as alkaline phosphatase (ALP), osteopontin (OPN), and collagen type I a1 chain (COL1A1), are believed to have value as bone development biomarkers (Shimoda et al., 2019). These markers are crucial in the differentiation of osteoblasts.

MicroRNAs (miRNAs) are small functional RNAs that are essential components of gene expression programmes that regulate numerous biological processes, such as cell death, differentiation, and proliferation (Bottini et al., 2017). MiRNAs function as important posttranscriptional regulators by binding to the 3′-untranslated region (3′-UTR) of their target mRNAs and suppressing target mRNA translation (Huang et al., 2019). The function of miRNAs in bone formation and bone growth has been extensively studied. A variety of miRNAs, including miR-224-5p (Ishiwata et al., 2020), miR-142a-5p (Yuan et al., 2021) and miR-34a (Hong et al., 2020), have been demonstrated to control the differentiation of bone precursor cells. Previous studies have shown that miR-452 plays different regulatory roles in different diseases. For example, miR-452-5p regulates the responsiveness of intestinal epithelial cells in inflammatory bowel diseases by inhibiting Mcl-1 expression (Deng et al., 2021). Yang et al. (2021) have indicated that miR-452 regulates C2C12 myoblast proliferation and differentiation by targeting ANGPT1. In terms of osteogenic differentiation, miR-452 is downregulated during the osteoblast differentiation of periodontal ligament stem cells, and overexpression of miR-452 suppresses the osteoblast differentiation by targeting the polycombgroup protein, BMI1(Mao et al., 2021). During osteogenic differentiation of mouse bone marrow mesenchymal stem cells, miR-452-3p is downregulated (Wang et al., 2018), but the specific molecular mechanism of its role in osteoblast differentiation remains unclear.

Osteoblasts differentiate directly into osteocytes during ossification. The Sry-related transcription factor Sox9, activator protein 1 (AP-1), runt-related transcription factor 2 (Runx2/Cbfa1), nuclear factor of activated T-cell cytoplasmic 1 (NFATc1) and twist are important in the process of osteogenic differentiation (Hsu, Chen & You, 2017). Smad4 is a key mediator of the transforming growth factor-β (TGF-β) pathway, which regulates cell proliferation, differentiation, and death (D’Inzeo et al., 2013). Previously, it was noted that smad4 is the key component of the TGF-β pathway and functions as a tumour suppressor (Yang & Yang, 2010). Defects in osteogenic differentiation facilitate the growth of tumours; therefore, a possible therapeutic technique is to stimulate this mechanism.

In this study, based on the above data, the involvement of miR-452-3p and Smad4 in osteoblast differentiation, was studied to determine whether these mediators could be used as a therapeutic target for osteoporosis.

## Materials and Methods

### Cell culture

The mouse osteoblast cell line (MC3T3-E1) was purchased from ScienCell (Carlsbad, CA, USA) and cultured in alpha-minimum essential medium (α-MEM; SH30265.01B; Hyclone, South Logan, UT, USA) containing 1% penicillin-streptomycin (Sigma-Aldrich, St. Louis, MO, USA), 10% foetal bovine serum (FBS). The cells were maintained at 37 °C in a humidified atmosphere containing 5% CO_2_. In order to induce osteoblast differentiation, the MC3T3-E1 cells were plated into six-well plate (10^6^ cells/well), cultured for 14 days in medium supplemented with 200 ng/mL BMP2 (Solarbio, Beijing, China), the medium was changed every 2 days.

### Cell transfection

MiR-452-3p mimic, inhibitor, and the corresponding negative controls (NC) were synthesized by GenePharma (Shanghai, China), and the target sequence of Smad4 small interfering RNA (siRNA) and siRNA of scrambled sequence were purchased from RIBOBIO (Guangzhou, China). Plasmids (10 nM) were transfected into MC3T3-E1 cells (six-well plate, 10^6^ cells/well) using the transfection reagent Lipofectamine RNAiMAX as directed by the manufacturer (13-778-075; Invitrogen, Carlsbad, CA, USA). The relevant primers were listed in Table 1.

**Table 1:** The sequence of primers, miRNA, siRNA, miRNA mimic and inhibitor used in the study.

Gene	Forward primer (5′-3′)	Reverse primer (5′-3′)
Smad4	ATCTATGCCCGTCTCTGGAGGT	CAGGMTGTTGGGAAAGTTGGC
ALP	CTACGCACCCTGTTCTGAGG	GGCCAAAGGGCAATAACTAG
Col1A1	GAAGCTTGGTCCTCTTGCTTG	CATTGCCTTTGTTTGCTGGG
OPN	GGACTGAGGTCAAAGTCTAGGAG	GGAATGCTCAAGTCTGTGTG
U6	CTCGCTTCGGCAGCACATATAC	GGAACGCTTCACGAATTTGC
GAPDH	CATCATCCCTGCATCCACTG	CAACCTGGTCCTCAGTGTAG
miR-452-3p	GCGAACTGTTTGCAGAGG	CAGTGGGTGTGGTGGAGT
Smad4	ATCTATGCCCGTCTCTGGAGGT	CAGGAATGTTGGGAAAGTTGGC
miR-452-3p mimic	AACUGUUUGCAGAGGAAACUG	
miR-452-3p mimic NC	UUCUCCGAACGUGUCACGUtt	
miR-452-3p inhibitor	AACUGUUUGCAGAGGAAACUGA	
miR-452-3p inhibitor NC	GGUUCGUACGUACACUGUUCA	
siRNA-Smad4	GUGUGCAGUUGGAAUGUAAUU	

### Quantitative real time-PCR (RT-PCR)

After 0, 7 and 14 days of osteoinduction, total RNA was extracted from MC3T3-E1 cells using TRIzol reagent (GenePharma). Utilizing a Bestar RT-qPCR system, cDNA was reverse transcribed from 50 ng of total RNA (DBI Bioscience, Shanghai, China). The cDNA template (20–100 ng) was utilized for RT-PCR with Bestar SYBR Green Master Mix (DBI Bioscience) according to the manufacturer’s instructions. RT-PCR was used to determine the levels of miR-452-3p and Smad4 in MC3T3-E1 cells. RT-PCR was performed to detect the expression of osteoblast differentiation-related genes, and three independent experiments were performed. The primers used for RT-PCR are listed in Table 1. The internal miRNA and mRNA controls were U6 and glyceraldehyde three-phosphate dehydrogenase (GAPDH), respectively. The gene expressions were quantified using the 2^−ΔΔCT^ method.

### Western blot analysis

Total protein was extracted using radioimmunoprecipitation assay (RIPA) buffer (pH 7.4) and protein concentration was determined by BCA kit (Solarbio, Beijing, China). 30 μg protein samples were separated by 10% SDS-PAGE, and then transferred to PVDF membranes (Millipore, Billerica, MA, USA), which were then blocked for 2 h at room temperature with 5% nonfat milk. Membranes were incubated with the anti-Smad4 primary antibody overnight (1:1,000, ab40759, Abcam, Cambridge, MA, USA) and were then incubated with a horseradish peroxidase-conjugated secondary antibody (approximately 1:5,000, goat IgG H&L anti-rabbit). Protein bands were identified with enhanced chemiluminescence reagents (Amersham Biosciences, Piscataway, NJ, USA). The intensity of the selected bands was quantified using Image J.

### ALP activity and Alizarin Red staining

ALP activity was assessed to evaluate the degree of differentiation in MC3T3-E1 cells. ALP activity was estimated using a commercial test kit (Jian Cheng Biotechnology, Nanjing, China) in BMP2-treated MC3T3-E1 cells (6-well plate) with siRNA-Smad4 and the miR-452-3p inhibitor or mimic transfection, and the absorbance was measured at 405 nm. Alizarin Red staining was conducted at room temperature for 30 min, and the cells were photographed under an inverted fluorescence microscope (Olympus, Tokyo, Japan).

### Plasmid construction and dual-luciferase reporter assay

Smad4 was predicted to be a target gene of miR-452-3p *via* TargetScan (http://www.targetscan.org/vert72/). A fragment of the Smad4 3′-UTR including the miR-452-3p binding sites was amplified and cloned into psiCHECK2 (Promega, Madison, WI, USA) to generate the PCR-specific Smad4 wild-type (WT) plasmid. For construction of the MUT Smad4 plasmid, a fragment that included mutated miR-452-3p binding sites was amplified and cloned into psiCHECK2. The MUT and WT Smad4 3′ UTR DNA sequences were generated by GenePharma. The Smad4 MUT or WT plasmid and the negative control (NC) or miR-452-3p mimic were transfected into 293T cells (six-well plate; 10^5^ cells/well) with Lipofectamine 2,000, and the cells in a 24-well plate were incubated overnight (Invitrogen). The luciferase activity was measured with a dual-luciferase reporter assay system (Promega, Madison, WI, USA) according to the product instructions.

### Statistical analysis

SPSS 16.0 software was used for statistical analysis. Data are represented as the mean ± SEM of at least three independent experiments. The difference between two groups was compared using two-tailed student’s t-test, or one-way analysis of variance (ANOVA) followed by the Scheffé test. *P* < 0.05 was considered to be statistically significant.

## Results

### Downregulation of miR-452-3p during osteoblast differentiation

**Figure 1:**
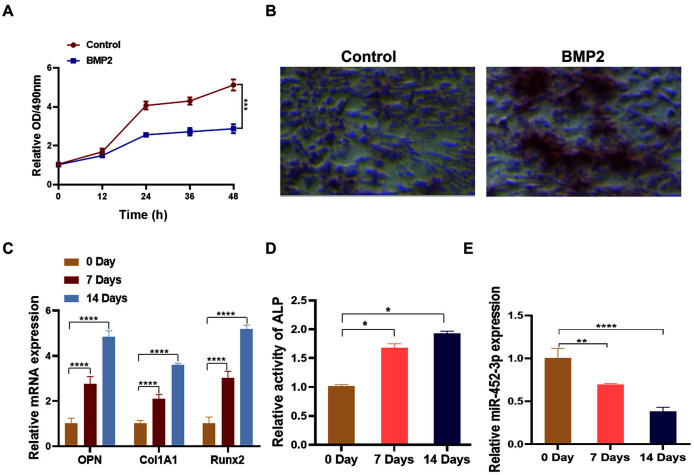
Downregulation of miR-452-3p during osteoblast differentiation. (A) The cell viability of MC3T3-E1 after 200 ng/mL BMP2 treatment for 0, 12, 24, 36, and 48 h was assessed using CCK-8 assay. (B) The number of calcium nodules was counted in 200 ng/mL BMP2-treated MC3T3-E1 cells by alizarin red staining, compared with untreated MC3T3-E1 group. (C) On days 0, 7, and 14, the mRNA levels of the osteoblast marker genes OPN, Col1A1, and Runx2 were assessed using by RT-PCR in induced MC3T3-E1 cells. (D) Alkaline phosphatase (ALP) activity in BMP2-treated MC3T3-E1 cells on days 0, 7 and 14 was measured using a commercial assay kit. (E) RT-PCR analysis of the expression of miR-452-3p in induced MC3T3-E1 cells. *N* = 3, *P < 0.05, **P < 0.01, ***P < 0.001, ****P < 0.0001.

To analyze the relationship between miR-452-3p and osteoblast differentiation, we first constructed the osteoblast differentiation cell model using BMP2. After being cultured in BMP2 medium for 14 days, the proliferation of MC3T3-E1 cells was significantly lower than that of the control group (Fig. 1A). Alizarine red staining was applied to further verify the successful osteoblast differentiation of MC3T3-E1 cells through BMP2. As shown in Fig. 1B, calcium nodules (orange red) significantly increased compared with the control group, indicating the successful establishment of osteoblast differentiation cell model. Then, compared with untreated cells, BMP2-induced cells showed increased expression of osteoblast marker genes (OPN, Col1A1, and Runx2) by RT-PCR analysis (Fig. 1C). Additionally, the activity of ALP was significantly enhanced under differentiation-inducing conditions for 7 and 14 days (Fig. 1D). Furthermore, we found that the mRNA expression level of miR-452-3p was significantly decreased compared with Day 0.

### miR-452-3p suppresses osteoblast differentiation

**Figure 2:**
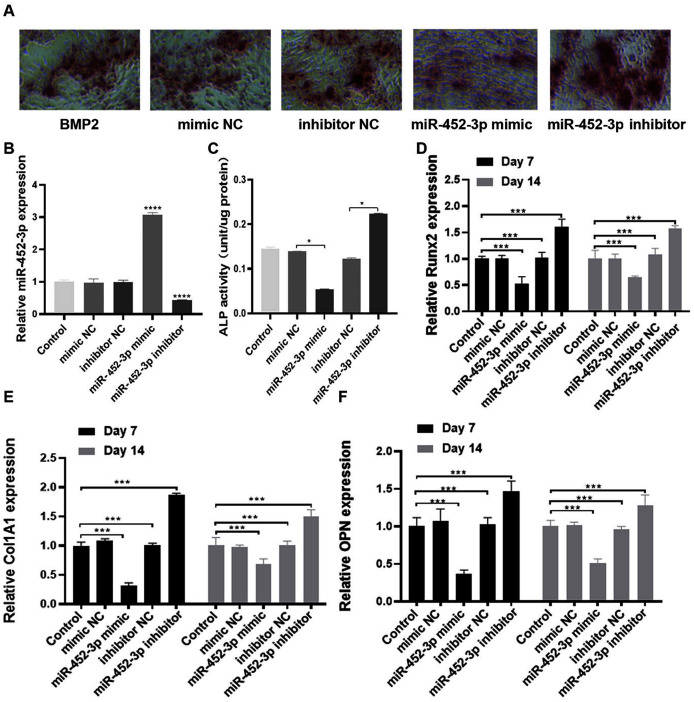
miR-452-3p suppresses osteoblast differentiation. (A) The number of calcium nodules was counted in 200 ng/mL BMP2-treated MC3T3-E1 cells by alizarin red staining under the transfection of mimic NC, inhibitor NC, miRNA-452-3p mimic, or miRNA-452-3p inhibitor. (B) RT-PCR analysis of the expression of miR-452-3p in 200 ng/mL BMP2-treated MC3T3-E1 cells under the transfection of mimic NC, inhibitor NC, miRNA-452-3p mimic, or miRNA-452-3p inhibitor. (C) Activity of ALP in 200 ng/mL BMP2-treated MC3T3-E1 cells under the transfection of mimic NC, inhibitor NC, miRNA-452-3p mimic, or miRNA-452-3p inhibitor. (D–F) RT-PCR analysis of the expression of Runx2 (D), Col1A1 (E), and OPN (F) in 200 ng/mL BMP2-treated MC3T3-E1 cells for 7 days and 14 days under the transfection of mimic NC, inhibitor NC, miRNA-452-3p mimic, or miRNA-452-3p inhibitor. *N* = 3, *P < 0.05, ***P < 0.001, ****P < 0.0001.

To elucidate the biological role of miR-452-3p in osteoblast differentiation, we investigated the gain and loss of function in MC3T3-E1 cells. The miR-452-3p inhibitor or mimic and the corresponding negative controls (NC) were transfected into MC3T3-E1 cells to overexpress or inhibit the mRNA expression of miR-452-3p (Fig. 2B). We first verified the effect of miR-452-3p on osteoblast differentiation by alizarin red staining, and the results showed that the transfection of miR-452-3p mimic significantly reduced the number of calcium nodules compared with the mimic NC. In contrast, miR-452-3p inhibitor increased the number of calcium nodules (Fig. 2A). Figure S1 showed the corresponding quantification results of alizarin red staining. The results further showed that ALP activity in differentiated MC3T3-E1 cells was inhibited after transfection with miR-452-3p mimics, but increased after transfection with miR-452-3p inhibitor (Fig. 2C). In addition, RT-PCR results (Figs. 2D–2F) showed that miR-452-3p mimic significantly reduced the expression of ossification related genes Runx2 (Fig. 2D), Col1A1 (Fig. 2E), and OPN (Fig. 2F) compared with the mimic NC, while miR-452-3p inhibitor significantly increased the levels of ossification related genes in MC3T3-E1 cells.

### Smad4 is a target gene of miR-452-3p

**Figure 3:**
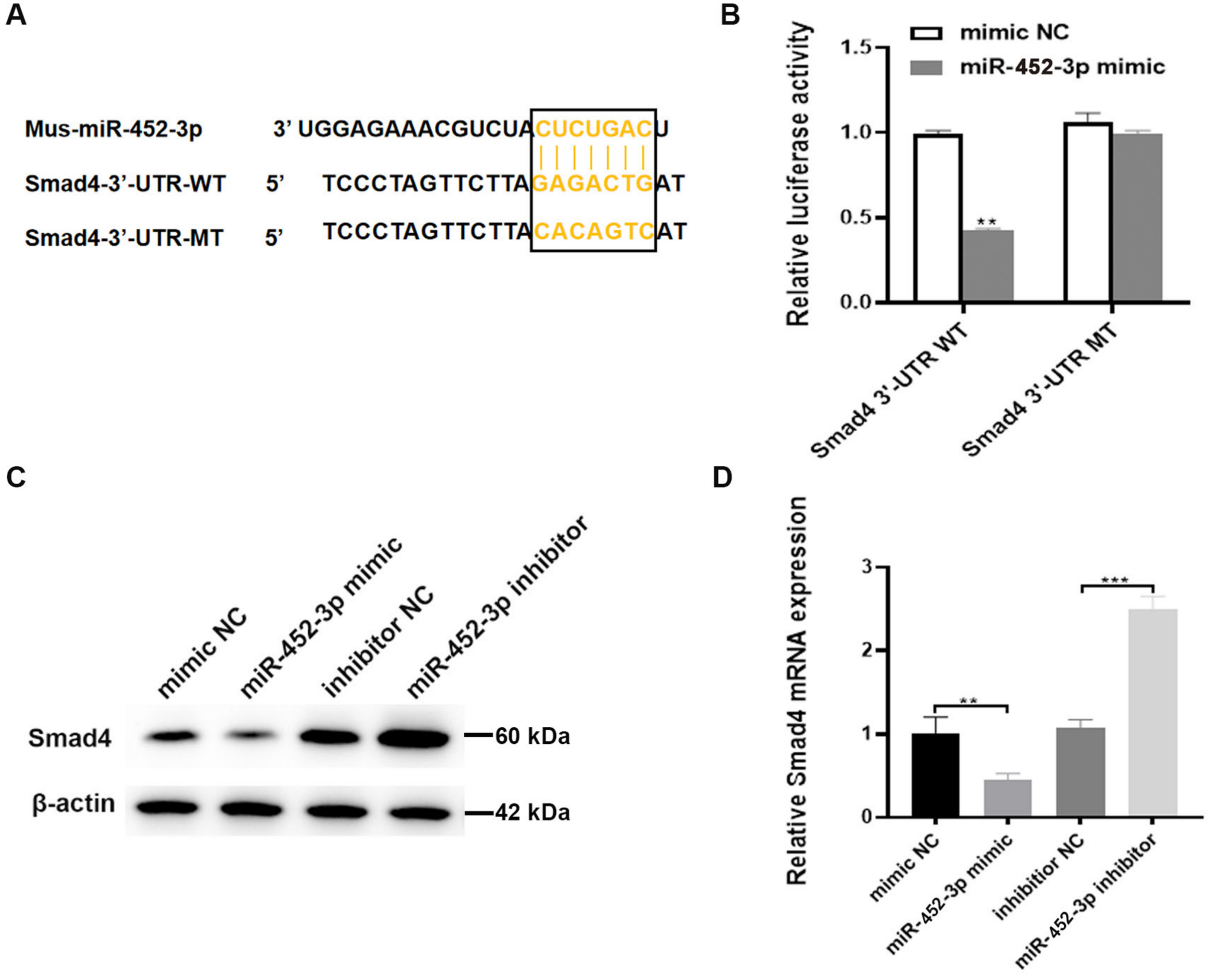
Smad4 is a target gene of miR-452-3p. (A) Schematic representation of the miR-452-3p site in Smad4-3′UTR. (B) The luciferase activity was assessed in MC3T3-E1 cells co-transfected with miRNA-452-3p and luciferase reporter plasmids containing the Smad4-3′UTR. Data are presented as the relative ratio of firefly to *Renilla* luciferase activity. (C, D) The levels of Smad4 protein (C) and mRNA (D) were measured by Western blotting and RT-PCR, respectively in MC3T3-E1 cells under the transfection of mimic NC, inhibitor NC, miRNA-452-3p mimic, or miRNA-452-3p inhibitor. *N* = 3, **P < 0.01, ***P < 0.001.

To explore the possible molecular mechanism of miR-452-3p in osteogenic differentiated MC3T3-E1 cells, the TargetScan database was used to search for possible targets of miR-452-3p. Smad4 was found to be a potential target of miR-452-3p (Fig. 3A). To verify the interaction between miR-452-3p and Smad4, the dual luciferase reporter gene assay was carried out in this study. The results showed that the overexpression of miR-452-3p significantly decreased the luciferase activity of Smad4-WT construct but not the MT construct (Fig. 3B). These results indicated that miR-452-3p could inhibit Smad4 mRNA translation by binding to a site located at 2229–2251 of the 3′-UTR. RT-PCR and Western blot analysis showed that Smad4 was significantly down-regulated in MC3T3-E1 cells transfected with miR-452-3p mimic, while Smad4 was up-regulated in MC3T3-E1 cells transfected with miR-452-3p inhibitor (Figs. 3C, 3D). Figure S2 shown the quantification of Western blot analysis. These results suggested that miR-452-3p may regulate Smad4 gene expression during osteoblast differentiation.

### miR-452-3p regulates osteoblast differentiation by targeting Smad4

**Figure 4:**
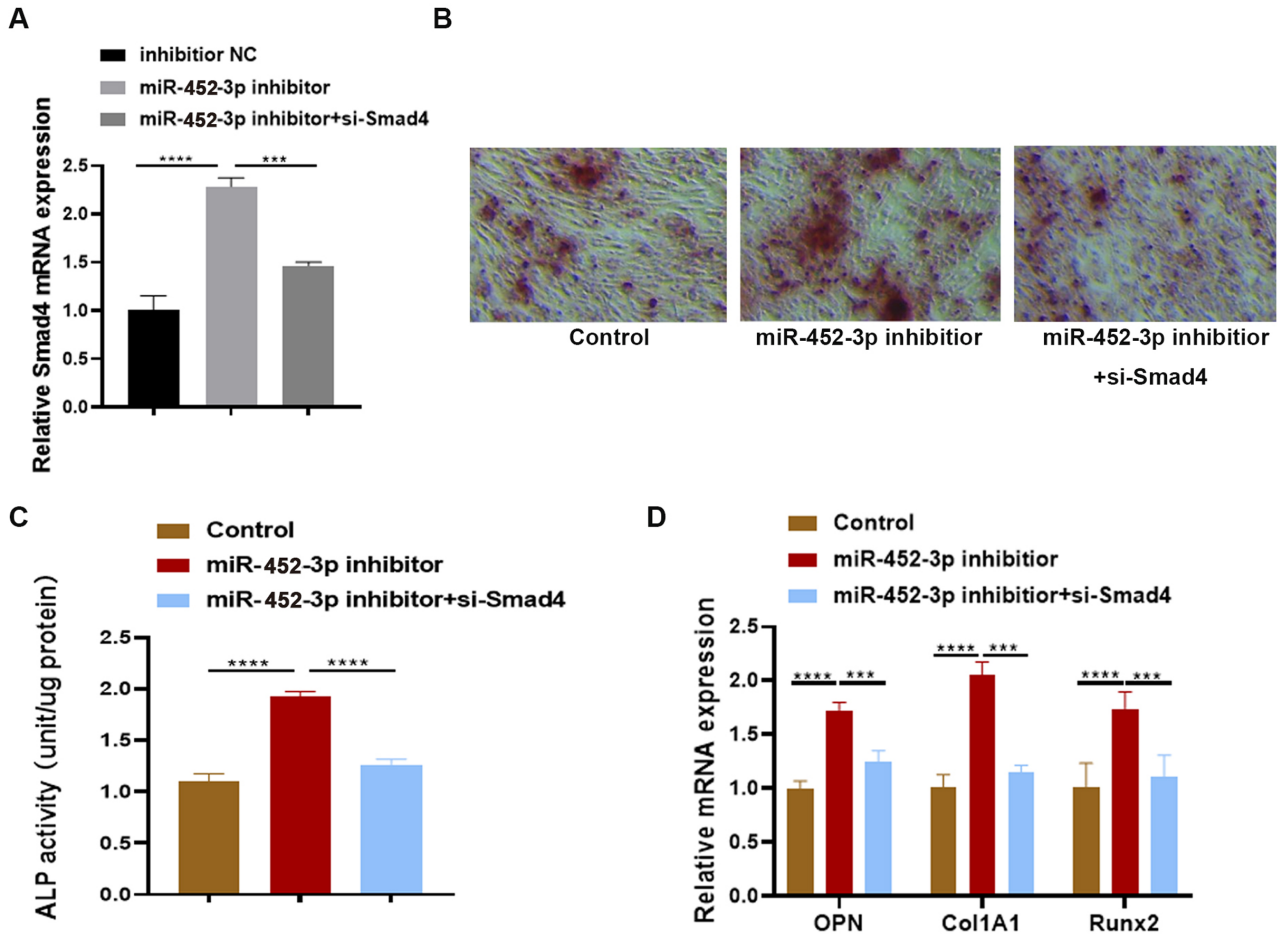
miR-452-3p regulates osteoblast differentiation by targeting Smad4. RT-PCR analysis of the expression of Smad4 in MC3T3-E1 cells under the transfection of inhibitor NC, miRNA-452-3p inhibitor and/or si-Smad4. (B) The number of calcium nodules was counted in MC3T3-E1 cells by alizarin red staining under the transfection of inhibitor NC, miRNA-452-3p inhibitor and/or si-Smad4. (C) Alizarin red staining for detecting cell calcification. (C) Activity of ALP in MC3T3-E1 cells under the transfection of inhibitor NC, miRNA-452-3p inhibitor and/or si-Smad4. (D) RT-PCR analysis of the expression of Runx2, Col1A1 and OPN in MC3T3-E1 cells under the transfection of inhibitor NC, miRNA-452-3p inhibitor and/or si-Smad4. *N* = 3, ***P < 0.001, ****P < 0.0001.

Huang et al. (2016) examined the effects of Smad4 gene on osteoblast differentiation and proliferation by transfecting the Smad4 overexpression plasmid (p-Smad4) and small interfering RNA (si-Smad4). The results showed that overexpression of Smad4 resulted in increased expression of osteoblast marker genes, could generate more ALP and more calcium deposition. In contrast, knockdown of Smad4 decreased the expression of osteoblast marker genes significantly, ALP activity and calcium deposition. These results suggested that Smad4 is a regulator of osteogenesis: overexpression of Smad4 promotes osteoblast differentiation, while downregulation of Smad4 inhibits osteogenesis (Huang et al., 2016). To further confirm whether Smad4 is involved in the osteoblast differentiation of miR-452-3p, we co-transfected Smad4 siRNA and miR-452-3p inhibitor into MC3T3-E1 cells. As shown in Fig. 4A, miR-452-3p inhibitor promoted the expression level of Smad4, and the inhibition of Smad4 (si-Smad4) reversed the expression of Smad4 enhanced by miR-452-3p inhibitor. As shown in Fig. 4B, si-Smad4 could reversed increased the number of calcium nodules by miR-452-3p inhibitor, by Alizarin red staining. Figure S3 showed the corresponding quantification results of alizarin red staining. ALP activity, and the mRNA expression of OPN, ALP and Col1A1 showed the same tendency (Figs. 4C, 4D). Overall, these results suggested that Smad4 was negatively regulated by miR-452-3p in the osteoblast differentiation.

## Discussion

In this study, the following findings were demonstrated by our results: (1) miR-452-3p is down-regulated during osteoblast differentiation; (2) miR-452-3p suppresses osteoblast differentiation; (3) Smad4 is a target gene of miR-452-3p; and (4) miR-452-3p regulates osteoblast differentiation by targeting Smad4.

Osteoclasts and osteoblasts are key players in retaining bone mass and are the major cells involved in bone remodelling (Zhan et al., 2019). Several miRNAs that participate in the differentiation of osteoblasts have been identified. For example, miR-497-5p (Zhao et al., 2020), microRNA-150-3p (Qiu et al., 2020), miR-708-5p (Wang et al., 2020), and microRNA-497-5p (Liu et al., 2020a) have been shown to promote this process, while miR-30 (Zhang et al., 2020a), miR-129-5p (Yin et al., 2020), miR-128-3p (Zhang et al., 2020b), and miR-17-5p have suppressive effects. In various cell types, miR-425-3p is differentially expressed (Li et al., 2020; Ma et al., 2019), but its function in osteogenesis has not been documented. The MC3T3-E1 pre-osteoblast cell line has been widely used for *in vitro* osteogenesis research (Lv et al., 2017). In the present research, we used these cells to investigate the mechanism by which osteoblast differentiation is regulated by miR-452-3p.

MiRNAs primarily function by interacting with their target mRNA’s 3′UTR and then negatively regulating gene expression (Jin et al., 2020). MiRNAs have been found to participate in the regulation of bone remodelling as major regulators (Sun et al., 2019). Smad4 is a key transcription factor in the TGF-β pathway, and malignant progression in prostate, colon and pancreatic carcinomas is associated with Smad4 loss (Demagny & De Robertis, 2016). *In vitro* studies have indicated that Smad4 interacts with transcription factors that affect MSC osteoblast differentiation, such as Runx2 and AP-1 (c-Fos-JunD) (Aspera-Werz et al., 2019). Osteoporosis associated with Snyder-Robinson syndrome is characterized by impaired osteoblast and osteoclast function (Albert et al., 2015). Smad4 is closely associated with the osteoblast differentiation factor Runx2. Smad4 deficiency impairs chondrocyte hypertrophy during mouse skeletal development through the Runx2 transcription factor (Yan et al., 2018). Promotion of Smad4 expression may be an important approach for the treatment of osteoporosis. Huang et al. (2016) have indicated that Smad4 is a regulator of osteogenesis: overexpression of Smad4 promotes osteoblast differentiation, while downregulation of Smad4 inhibits osteogenesis. In our research, we found that by downregulating the expression of smad4, miR-452-3p suppressed osteoblast differentiation. However, it is necessary to verify the clinical significance of this result in connection with osteoporosis. Furthermore, while there is evidence that the Smad4 signalling pathway plays an important role in osteoblast differentiation, the specific mechanism through which miR-452-3p regulates Smad4 during osteogenesis requires further study.

## Conclusion

The findings of our research show for the first time that by suppressing smad4 expression, miR-452-3p negatively regulates osteoblast differentiation. Therefore, therapeutic inhibition of miR-452-3p can potentially support the growth of bone and can be an effective treatment for orthopaedic conditions, for example, osteoporosis.

## Supplemental Information

10.7717/peerj.12228/supp-1 Supplemental Information 1 All raw data.

10.7717/peerj.12228/supp-2 Supplemental Information 2 The corresponding quantification results of alizarin red staining.

10.7717/peerj.12228/supp-3 Supplemental Information 3 The quantification of Western Blot analysis.

10.7717/peerj.12228/supp-4 Supplemental Information 4 The corresponding quantification results of alizarin red staining.

## Abbreviations

BMP2: bone morphogenetic protein 2
GAPDH: glyceraldehyde-3-phosphate dehydrogenase
MUT: mutated
ALP: alkaline phosphatase
miR-452-3p: microRNA-452-3p
OPN: osteopontin
siRNA: small interfering RNA
Smad4: SMAD Family Member 4
UTR: untranslated region
WT: wild-type
NC: negative control
COL1A1: collagen type I ɑ1 chain

## Additional Information and Declarations

### Data Availability

The following information was supplied regarding data availability: The raw measurements are available in the Supplementary File.

## References

[ref-1] Albert JS, Bhattacharyya N, Wolfe LA, Bone WP, Maduro V, Accardi J, Adams DR, Schwartz CE, Norris J, Wood T, Gafni RI, Collins MT, Tosi LL, Markello TC, Gahl WA, Boerkoel CF (2015). Impaired osteoblast and osteoclast function characterize the osteoporosis of Snyder-Robinson syndrome. Orphanet Journal of Rare Diseases.

[ref-2] Aspera-Werz R, Chen T, Ehnert S, Zhu S, Fröhlich T, Nussler AK (2019). Cigarette smoke induces the risk of metabolic bone diseases: transforming growth factor beta signaling impairment via dysfunctional primary cilia affects migration, proliferation, and differentiation of human mesenchymal stem cells. International Journal of Molecular Sciences.

[ref-3] Bottini S, Hamouda-Tekaya NH, Mategot R, Zaragosi LE, Audebert S, Pisano S, Grandjean V, Mauduit C, Benahmed M, Barbry P, Repetto E, Trabucchi M (2017). Post-transcriptional gene silencing mediated by microRNAs is controlled by nucleoplasmic SFPQ. Nature Communications.

[ref-4] D’Inzeo S, Nicolussi A, Nardi F, Coppa A (2013). Effects of the Smad4 C324Y mutation on thyroid cell proliferation. International Journal of Oncology.

[ref-5] Demagny H, De Robertis EM (2016). Point mutations in the tumor suppressor Smad4/DPC4 enhance its phosphorylation by GSK3 and reversibly inactivate TGF-β signaling. Molecular & Cellular Oncology.

[ref-6] Deng M, Hu J, Tong R, Guo H, Liu Y (2021). miR-452-5p regulates the responsiveness of intestinal epithelial cells in inflammatory bowel disease through Mcl-1. Experimental and Therapeutic Medicine.

[ref-7] Hong M, Zhang XB, Xiang F, Fei X, Ouyang XL, Peng XC (2020). MiR-34a suppresses osteoblast differentiation through glycolysis inhibition by targeting lactate dehydrogenase-A (LDHA). In Vitro Cellular & Developmental Biology-Animal.

[ref-8] Hsu WH, Chen CM, You LR (2017). COUP-TFII is required for morphogenesis of the neural crest-derived tympanic ring. Scientific Reports.

[ref-9] Huang C, Geng J, Wei X, Zhang R, Jiang S (2016). MiR-144-3p regulates osteogenic differentiation and proliferation of murine mesenchymal stem cells by specifically targeting Smad4. FEBS Letters.

[ref-10] Huang W, Huang Y, Gu J, Zhang J, Yang J, Liu S, Xie C, Fan Y, Wang H (2019). miR-23a-5p inhibits cell proliferation and invasion in pancreatic ductal adenocarcinoma by suppressing ECM1 expression. American Journal of Translational Research.

[ref-11] Ishiwata S, Iizuka H, Sonoda H, Tsunoda D, Tajika Y, Chikuda H, Koibuchi N, Shimokawa N (2020). Upregulated miR-224-5p suppresses osteoblast differentiation by increasing the expression of Pai-1 in the lumbar spine of a rat model of congenital kyphoscoliosis. Molecular and Cellular Biochemistry.

[ref-12] Jin L, Cai Q, Wang S, Wang S, Wang J, Quan Z (2020). Long noncoding RNA PVT1 promoted gallbladder cancer proliferation by epigenetically suppressing miR-18b-5p via DNA methylation. Cell Death & Disease.

[ref-13] Learmonth I, Young C, Rorabeck C (2007). The operation of the century: total hip replacement. The Lancet.

[ref-14] Li J, Tu J, Gao H, Tang L (2020). MicroRNA-425-3p inhibits myocardial inflammation and cardiomyocyte apoptosis in mice with viral myocarditis through targeting TGF-β1. Immunity, Inflammation and Disease.

[ref-15] Liu J, Wang X, Song M, Du J, Yu J, Zheng W, Zhang C, Wang Y (2020a). Smurf2MiR-497-5p regulates Osteo/Odontogenic differentiation of stem cells from apical papilla via the Smad signaling pathway by targeting. Frontiers in Genetics.

[ref-16] Liu X, Li Z, Liu H, Zhu Y, Xia D, Wang S, Gu R, Zhang P, Liu Y, Zhou Y (2020b). Flufenamic acid inhibits adipogenic differentiation of mesenchymal stem cells by antagonizing the PI3K/AKT signaling pathway. Stem Cells International.

[ref-17] Lv M, Liu Y, Xiao TH, Jiang W, Lin BW, Zhang XM, Lin YM, Xu ZS (2017). viaGYY4137 stimulates osteoblastic cell proliferation and differentiation an ERK1/2-dependent anti-oxidant mechanism. American Journal of Translational Research.

[ref-18] Ma Y, Yuwen D, Chen J, Zheng B, Gao J, Fan M, Xue W, Wang Y, Li W, Shu Y, Xu Q, Shen Y (2019). Exosomal transfer of cisplatin-induced miR-425-3p confers cisplatin resistance in NSCLC through activating autophagy. International Journal of Nanomedicine.

[ref-19] Mao T, Li J, Peng R, Liu R, Peng Y (2021). miR-452 negatively regulates osteoblast differentiation in periodontal ligament stem cells by targeting the polycombgroup protein, BMI1. Tropical Journal of Pharmaceutical Research April.

[ref-20] Oh J, Ahn B, Karadeniz F, Kim J, Lee J, Seo Y, Kong CS (2019). EckloniaPhlorofucofuroeckol a from edible brown alga enhances osteoblastogenesis in bone marrow-derived human mesenchymal stem cells. Marine Drugs.

[ref-21] Qiu M, Zhai S, Fu Q, Liu D (2020). Bone marrow mesenchymal stem cells-derived exosomal microRNA-150-3p promotes osteoblast proliferation and differentiation in osteoporosis. Human Gene Therapy.

[ref-22] Raic A, Friedrich F, Kratzer D, Bieback K, Lahann J, Thedieck CL (2019). Potential of electrospun cationic BSA fibers to guide osteogenic MSC differentiation via surface charge and fibrous topography. Scientific Reports.

[ref-23] Ruan C, Hu N, Ma Y, Li Y, Liu J, Zhang X, Pan H (2017). The interfacial pH of acidic degradable polymeric biomaterials and its effects on osteoblast behavior. Scientific Reports.

[ref-24] Shimoda A, Sawada S, Sasaki Y, Akiyoshi K (2019). Exosome surface glycans reflect osteogenic differentiation of mesenchymal stem cells: profiling by an evanescent field fluorescence-assisted lectin array system. Scientific Reports.

[ref-25] Sun L, Liu M, Luan S, Shi Y, Wang Q (2019). MicroRNA-744 promotes carcinogenesis in osteosarcoma through targeting LATS2. Oncology Letters.

[ref-26] Wang A, Ren M, Song Y, Wang X, Wang Q, Yang Q, Liu H, Du Z, Zhang G, Wang J (2018). MicroRNA expression profiling of bone marrow mesenchymal stem cells in steroid-induced osteonecrosis of the femoral head associated with osteogenesis. Medical Science Monitor.

[ref-27] Wang R, Feng Y, Xu H, Huang H, Zhao S, Wang Y, Li H, Cao J, Xu G, Huang S (2020). Synergistic effects of miR-708-5p and miR-708-3p accelerate the progression of osteoporosis. Journal of International Medical Research.

[ref-28] Yan J, Li J, Hu J, Zhang L, Wei C, Sultana N, Cai X, Zhang W, Cai C-L (2018). Smad4 deficiency impairs chondrocyte hypertrophy via the Runx2 transcription factor in mouse skeletal development. Journal of Biological Chemistry.

[ref-29] Yang G, Yang X (2010). Smad4-mediated TGF-beta signaling in tumorigenesis. International Journal of Biological Sciences.

[ref-30] Yang L, Qi Q, Wang J, Song C, Wang Y, Chen X, Chen H, Zhang C, Hu L, Fang X (2021). MiR-452 regulates C2C12 myoblast proliferation and differentiation targeting. Frontiers in Genetics.

[ref-31] Yin C, Tian Y, Yu Y, Yang C, Su P, Zhao Y, Wang X, Zhang K, Pei J, Li D, Chen Z, Zhang Y, Miao Z, Qian A (2020). miR-129-5p inhibits bone formation through TCF4. Frontiers in Cell and Developmental Biology.

[ref-32] Yuan H, Li M, Feng X, Zhu E, Wang B (2021). miR-142a-5p promoted osteoblast differentiation via targeting nuclear factor IA. Journal of Cellular Physiology.

[ref-33] Zhan Y, Liang J, Tian K, Che Z, Wang Z, Yang X, Su Y, Lin X, Song F, Zhao J, Xu J, Liu Q, Zhou B (2019). Vindoline inhibits RANKL-induced osteoclastogenesis and prevents ovariectomy-induced bone loss in mice. Frontiers in Pharmacology.

[ref-34] Zhang L, Li G, Wang K, Wang Y, Dong J, Wang H, Xu L, Shi F, Cao X, Hu Z, Zhang S (2020a). MiR-30 family members inhibit osteoblast differentiation by suppressing Runx2 under unloading conditions in MC3T3-E1 cells. Biochemical and Biophysical Research Communications.

[ref-35] Zhang W, Zhu Y, Chen J, Wang J, Yao C, Chen C (2020b). Mechanisms of miR‐128‐3p in inhibiting osteoblast differentiation from bone marrow‐derived mesenchymal stromal cells. Molecular Medicine Reports.

[ref-36] Zhao H, Yang Y, Wang Y, Feng X, Deng A, Ou Z, Chen B (2020). MicroRNA-497-5p stimulates osteoblast differentiation through HMGA2-mediated JNK signaling pathway. Journal of Orthopaedic Surgery and Research.

