# Supplementary figures and images for: miR-452-3p inhibited osteoblast differentiation by targeting Smad4
Source: PeerJ. 2021 Sep 28;9:e12228. doi: 10.7717/peerj.12228 (PMC8485836; doi:10.7717/peerj.12228)

Fig 3C Smad4


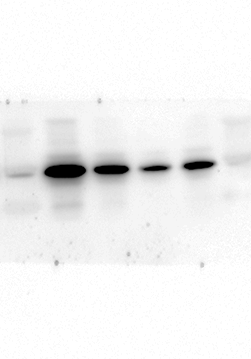


Fig 3C β-actin


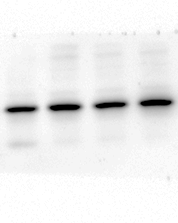

Supplement: Supplemental Information 1 [file peerj-09-12228-s001.zip › Raw data/WB/WB.docx]

Fig 3C Smad4

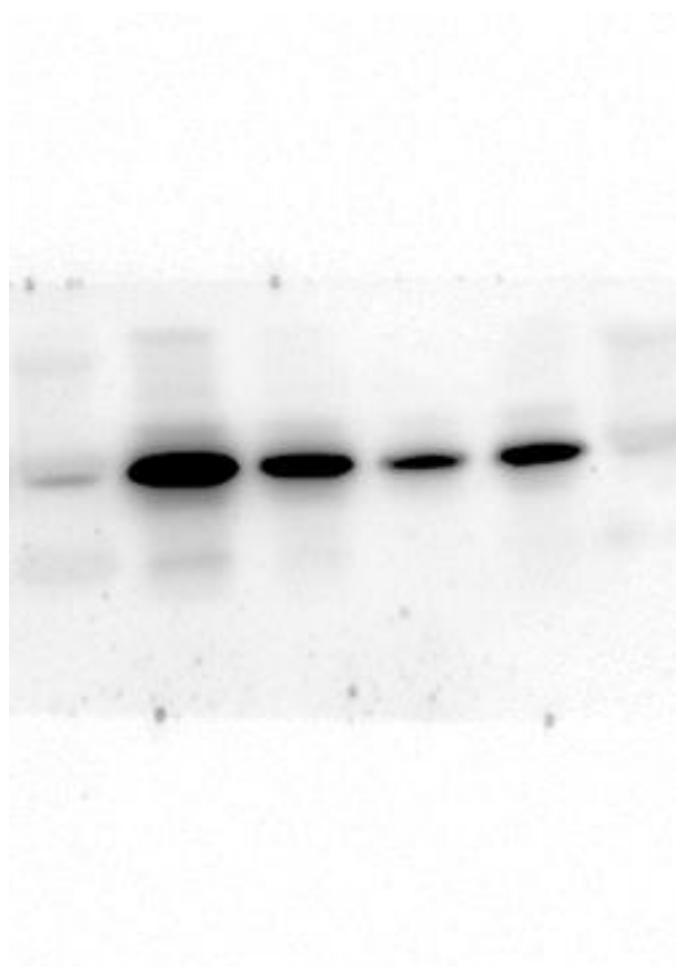

Fig 3C  $\beta$ -actin

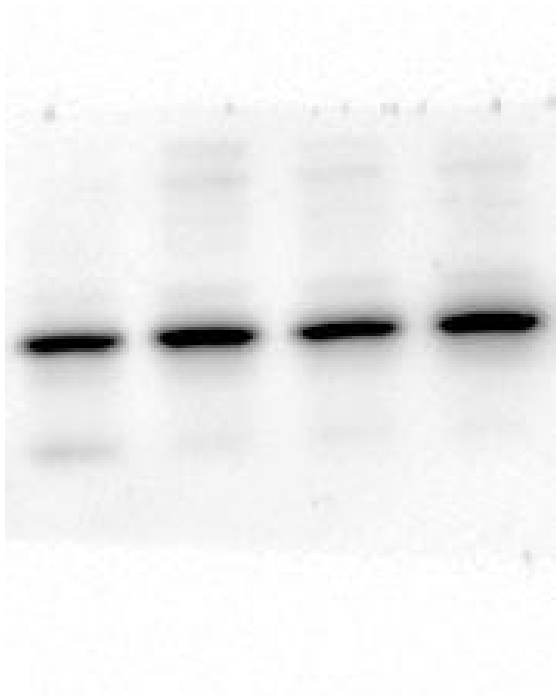

Supplement: Supplemental Information 1 [file peerj-09-12228-s001.zip › Raw data/WB/WB.pdf]

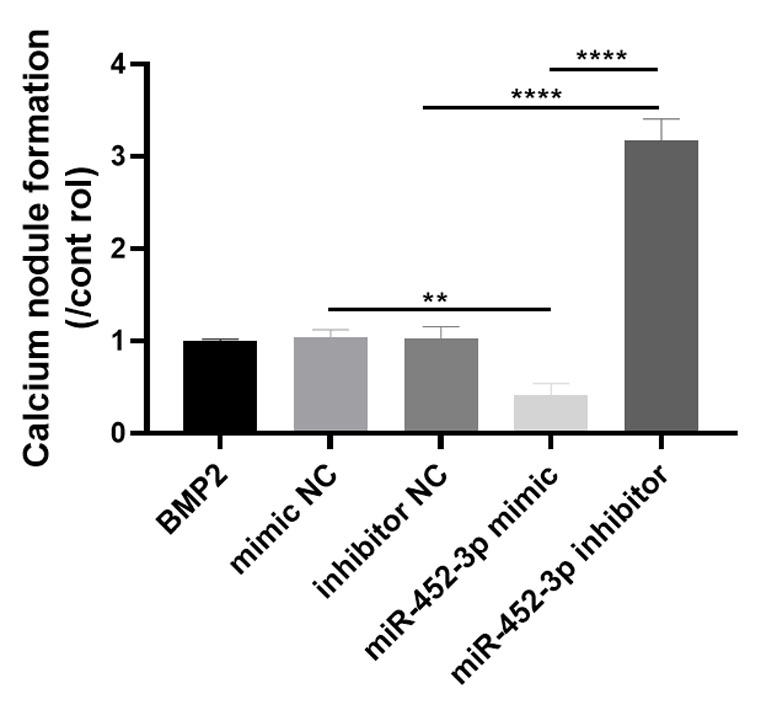

Supplement: Supplemental Information 2 [file peerj-09-12228-s002.jpg]

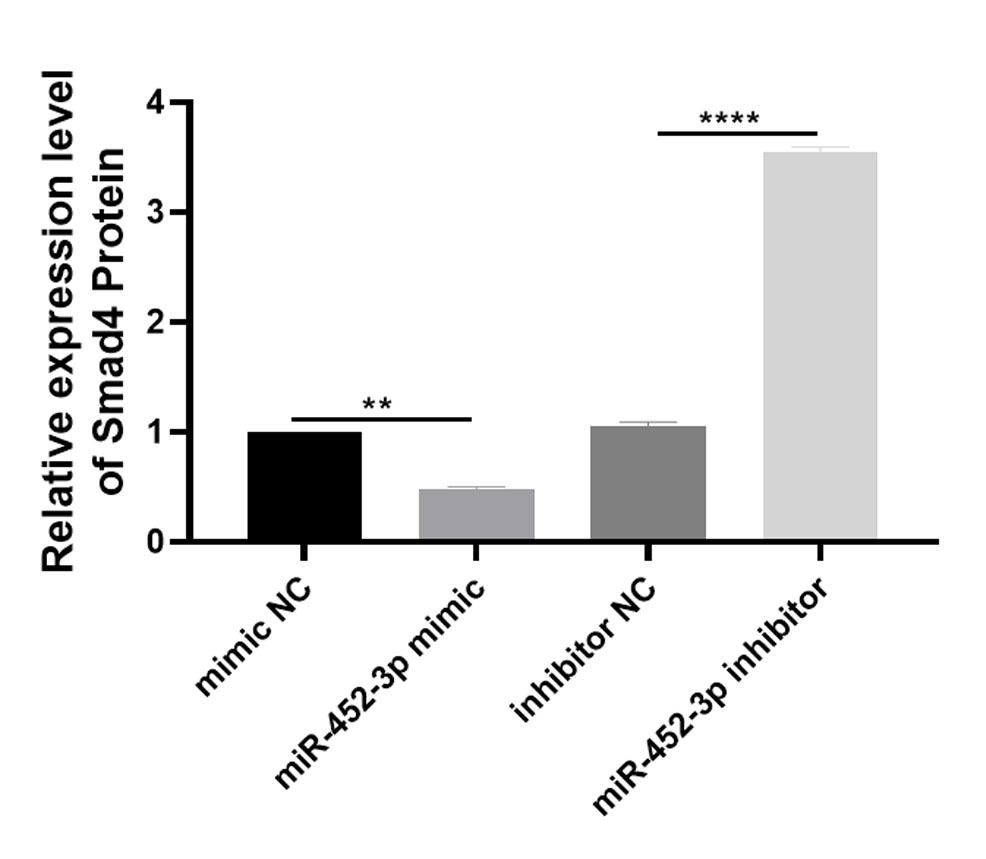

Supplement: Supplemental Information 3 [file peerj-09-12228-s003.jpg]

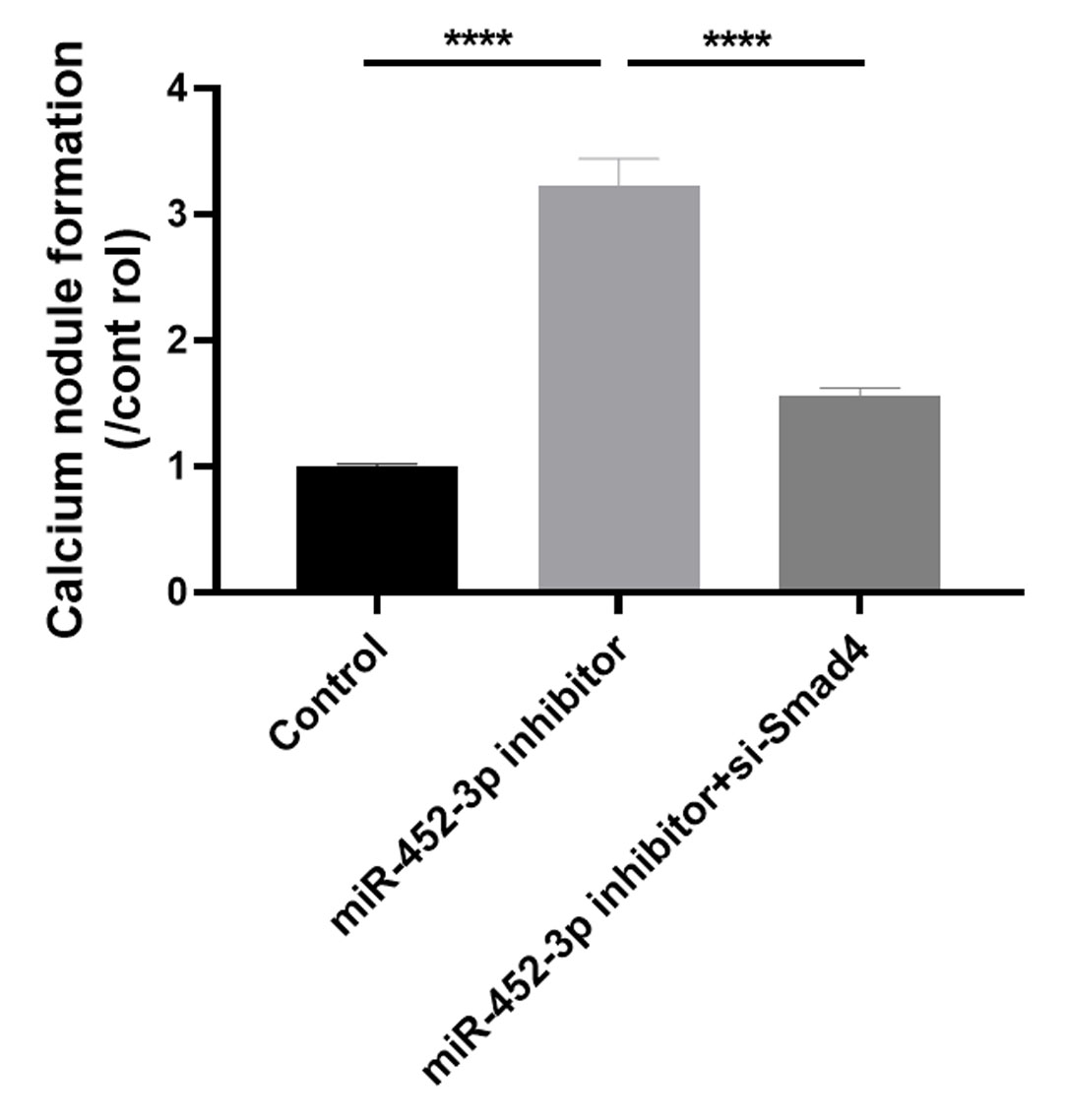

Supplement: Supplemental Information 4 [file peerj-09-12228-s004.jpg]
